# A preliminary analysis of clinical characteristics of patient with alcohol use disorder and suicidal ideation

**DOI:** 10.1192/j.eurpsy.2024.402

**Published:** 2024-08-27

**Authors:** R. F. Palma-Alvarez, A. Rios-Landeo, G. Ortega-Hernandez, E. Ros-Cucurull, C. Daigre, M. Perea-Ortueta, L. Grau-Lopez, J. A. Ramos-Quiroga

**Affiliations:** ^1^ Hospital Universitari Vall d’Hebron; ^2^Universitat Autònoma de Barcelona, Barcelona, Spain

## Abstract

**Introduction:**

Suicidal behaviors are frequently observed among patients with substance use disorder, including suicidal ideation (SI) (1). Alcohol use disorder (AUD) is one of the most prevalent addictions and may be related to suicidal behaviors (2,3). However, the association between AUD and SI requires a deeper analysis which includes several clinical features observed among AUD patients.

**Objectives:**

To analyze the clinical characteristics and features associated with lifetime SI among patients who had AUD.

**Methods:**

This is a cross-sectional study performed in an outpatient center for addiction treatment in patients seeking treatment who met the criteria for AUD between 01/01/2010 and 12/31/2021. Patients were evaluated with an ad-hoc questionnaire and the European addiction severity index (EuropASI). SI was evaluated by using the item for SI in EuropASI.

**Results:**

From a potential sample of n=3729 patients, only n=1082 (73.8% males; mean age 42.82±12.51) met inclusion criteria and had data for the current analysis. Lifetime SI was present in 50.9% of the AUD patients. Several clinical features were related to SI, including: sex differences, any type of lifetime abuse, polyconsumption, benzodiazepine use disorder, any psychiatric diagnosis aside of SUD, and higher addiction severity according to the EuropASI (See table)

**Image:**

**Figure:**
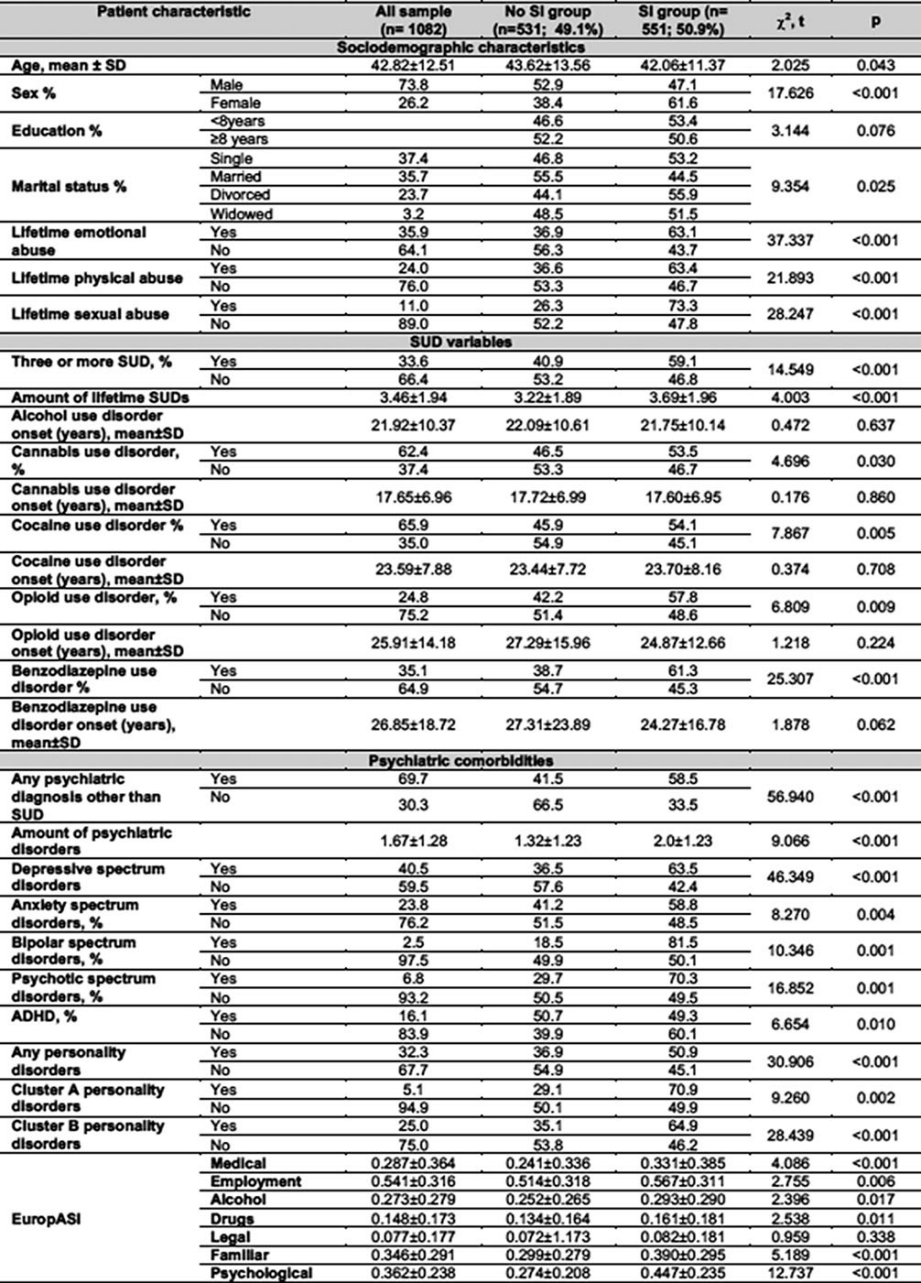

**Conclusions:**

SI among AUD patients is related to several clinical features which indicate a higher addiction severity, more polyconsumption, and a higher prevalence of psychiatric comorbidities. These findings may contribute to the understanding of suicidal behaviors in AUD patients but it is required further investigations, including longitudinal studies.

REFERENCES

1. Rodríguez-Cintas L, et al. Factors associated with lifetime suicidal ideation and suicide attempts in outpatients with substance use disorders. *Psychiatry Res.* 2018;262:440-445. doi:10.1016/j.psychres.2017.09.02

2. MacKillop J, et al. Hazardous drinking and alcohol use disorders. *Nat Rev Dis Primers.* 2022;8(1):80. doi:10.1038/s41572-022-00406-1 3. Darvishi N, et al. Alcohol-related risk of suicidal ideation, suicide attempt, and completed suicide: a meta-analysis [published correction appears in PLoS One. 2020;15(10):e0241874]. *PLoS One.* 2015;10(5):e0126870. doi:10.1371/journal.pone.0126870

**Disclosure of Interest:**

R. Palma-Alvarez Speakers bureau of: RFPA has received speaker honorariums from Angelini, Cassen Recordati, Exeltis, Lundbeck, MSD, Rubió, Servier, and Takeda., A. Rios-Landeo: None Declared, G. Ortega-Hernandez Speakers bureau of: GOH has received speaker honorariums from Rubió., E. Ros-Cucurull Speakers bureau of: ERC has received speaker honorariums from Janssen-Cilag, Lundbeck, Otsuka, Pfizer, Lilly, Servier, Rovi, Juste., C. Daigre: None Declared, M. Perea-Ortueta: None Declared, L. Grau-Lopez Speakers bureau of: LGL has received fees to give talks for Janssen-Cilag, Lundbeck, Servier, Otsuka, and Pfizer., J. Ramos-Quiroga Speakers bureau of: JARQ has been on the speakers’ bureau and/or acted as consultant for Janssen-Cilag, Novartis, Shire, Takeda, Bial, Shionogi, Sincrolab, Novartis, BMS, Medice, Rubió, Uriach and Raffo.

